# Mitochondrial pathology in progressive cerebellar ataxia

**DOI:** 10.1186/s40673-015-0035-x

**Published:** 2015-12-04

**Authors:** David Bargiela, Priya Shanmugarajah, Christine Lo, Emma L. Blakely, Robert W. Taylor, Rita Horvath, Stephen Wharton, Patrick F. Chinnery, Marios Hadjivassiliou

**Affiliations:** Academic Department of Neurosciences, Royal Hallamshire Hospital, Glossop Road, Sheffield, S10 2JF UK; Institute of Genetic Medicine, Newcastle University, Newcastle upon Tyne, UK; Wellcome Trust Centre for Mitochondrial Research, Institute of Neuroscience, Newcastle University, Newcastle upon Tyne, UK; Institute of Human Genetics, Newcastle University, Newcastle upon Tyne, UK; Department of Histopathology, Royal Hallamshire Hospital, Sheffield, UK

**Keywords:** Ataxia, Mitochondrial disease, Muscle mitochondria, Histopathology, Genetics

## Abstract

**Background:**

Mitochondrial disease can manifest as multi-organ disorder, often with neurological dysfunction. Cerebellar ataxia in isolation or in combination with other features can result from mitochondrial disease yet genetic testing using blood DNA is not sufficient to exclude this as a cause of ataxia. Muscle biopsy is a useful diagnostic tool for patients with ataxia suspected of mitochondrial disease. Our aim was to determine specific patient selection criteria for muscle biopsy to see how frequent mitochondrial mutations are responsible for progressive ataxia. We performed a two centre retrospective review of patients with unexplained progressive ataxia who underwent muscle biopsy for suspected mitochondrial disease between 2004 and 2014 (Sheffield and Newcastle Ataxia Centres).

**Results:**

A total of 126 patients were identified; 26 assessed in Newcastle and 100 in Sheffield. Twenty-four patients had pure ataxia and 102 had ataxia with additional features. The total number of patients with histologically suspected and/or genetically confirmed mitochondrial disease was 29/126 (23 %).

**Conclusions:**

A large proportion of patients (23 %) with progressive ataxia who underwent muscle biopsy were found to have features of mitochondrial dysfunction, with molecular confirmation in some. Muscle biopsy is a helpful diagnostic tool for mitochondrial disease in patients with progressive ataxia.

## Background

Mitochondrial disease represents a heterogeneous group of disorders caused by a primary defect of mitochondrial respiratory chain dysfunction. They present at any age and are a result of mutations either in the nuclear or mitochondrial DNA (mtDNA) [1]. Mitochondrial disease can manifest as a multi-organ disorder frequently presenting with neurological dysfunction with deafness, myopathy, ataxia, ptosis, ophthalmoplegia and retinitis pigmentosa amongst the most common neurological features encountered [2]. Many genetically-characterised mitochondrial disorders manifest with a combination of clinical features comprising distinct syndromes such as Kearns-Sayre syndrome (KSS) [3], chronic progressive external ophthalmoplegia (CPEO) [3], mitochondrial encephalopathy, lactic acidosis and stroke like episodes (MELAS) [4], myoclonic epilepsy with ragged red fibres (MERRF) [5], neurogenic weakness with ataxia and retinitis pigmentosa (NARP) and Leigh syndrome (LS) [6]. Even within these syndromes, clinical variability may exist and not all patients fit into a distinct clinical phenotype.

More of a diagnostic challenge is the clinical scenario where a limited number of such features are present with or without a family history. This is particularly true in patients presenting with cerebellar ataxia. Despite ataxia being a recognised feature in some of the well-characterised mitochondrial syndromes such as MELAS, MERRF, NARP, *POLG*-related ataxia neuropathy spectrum (ANS) [7] that includes mitochondrial recessive ataxia syndrome (MIRAS) and sensory ataxia neuropathy dysarthria and ophthalmoplegia (SANDO), genetic testing for the above mutations is not always positive. This is because the specific mutation may not have been tested for, or because the mtDNA mutation is not detectable in blood. In this context, muscle biopsy can be a useful investigation in the diagnostic work up of suspected cases as it can demonstrate histological and histochemical abnormalities suggestive of an underlying mitochondrial biochemical defect [8]. There are, however no specific guidelines for selecting patients with progressive ataxia for a muscle biopsy. The role and diagnostic yield of a muscle biopsy in any patient with progressive ataxia of undetermined cause requires clarification.

A large two-centre collaboration between the Sheffield and Newcastle Ataxia Centres and the Newcastle Highly Specialised Service Mitochondrial Diagnostic laboratory led to a retrospective review of clinical, genetic and muscle histological characteristics of all patients with progressive ataxia of undetermined cause who underwent muscle biopsy to specifically investigate a possible mitochondrial aetiology between 2004 and 2014. There are only 4 such ataxia centres in the UK, receiving referrals from all over the UK. The diagnostic yield for such referrals where a diagnosis has not been reached by the referring centre is approximately 50 %. The aim of this retrospective review was twofold: firstly to establish the frequency of mitochondrial disease as the cause of progressive ataxia and secondly to identify any additional clinical characteristics that are likely to indicate which patients require muscle biopsy as part of the diagnostic work-up.

## Results

### Clinical characteristics

A total of 126 patients were included; 26 assessed in Newcastle and 100 in Sheffield. There were no statistically significant differences in the frequency of major clinical features between the Newcastle and Sheffield cohorts (see Table 1) hence the results are presented for the group of 126 patients as a whole. The mean age at the time of the muscle biopsy was 52 years. The male to female ratio was 72 to 54. Patients displaying the ‘ataxia plus’ phenotype dominated the group with 102 having ‘ataxia plus’ vs. 24 having ‘pure ataxia’ (see Table 2). Family history was present in 50/126 (40 %) of patients. Of the patients with a family history, 21/50 (42 %) had autosomal recessive inheritance, 14/50 (28 %) autosomal dominant and 15/50 (30 %) maternal.

**Table 1:** Clinical characteristics of the Sheffield and Newcastle cohorts

	Newcastle	Sheffield	All
	*n* = 26	*n* = 100	*n* = 126
Clinical			
Mean age at muscle biopsy (range)	50 (22–76)	53 (18–78)	52 (18–78)
Sex (M:F)	14:12	58:42	72:54
Family history	8 (31 %)	42 (42 %)	50 (40 %)
Phenotype (pure ataxia: ataxia plus)	4:22	20:80	24:102

**Table 2:** Additional clinical features in patients with histologically suspected or genetically confirmed mitochondrial disease

	Patient groups		
	Histologically suspected mitochondrial disease without genetic confirmation	Genetically confirmed mitochondrial disease	Unlikely mitochondrial disease remaining patients
Total	21/126 (17 %)	11/126 (9 %)	94/126 (75 %)
Family History	12/21 (57 %)	2/11 (18 %)	36/94 (38 %)
Autosomal recessive	5/21 (24 %)	0/11 (0 %)	16/94 (17 %)
Autosomal dominant	3/21 (14 %)	1/11 (9 %)	10/94 (11 %)
Maternal	4/21 (19 %)	1/11 (9 %)	10/94 (11 %)
No Family History	9/21 (43 %)	9/11 (82 %)	58/94 (62 %)
Pure Ataxia	3/21 (14 %)	0/11 (0 %)	21/94 (22 %)
Ataxia Plus (Associated features)	18/21 (86 %)	11/11 (100 %)	73/94 (78 %)
Neurological			
Deafness	3/21 (14 %)	5/11 (45 %)	19/94 (20 %)
Dysphagia	0/21 (0 %)	2/11 (18 %)	0/94 (0 %)
Developmental problems	1/21 (5 %)	2/11 (18 %)	11/94 (12 %)
Cognitive deficits	2/21 (10 %)	2/11 (18 %)	6/94 (6 %)
Dystonia	2/21 (10 %)	0/11 (0 %)	2/94 (2 %)
Myoclonus	2/21 (10 %)	3/11 (27 %)	14/94 (15 %)
Epilepsy	2/21 (10 %)	2/11 (18 %)	12/94 (13 %)
Peripheral neuropathy	5/21 (24 %)	1/11 (9 %)	21/94 (22 %)
Spastic paraparesis	7/21 (33 %)	2/11 (18 %)	7/94 (7 %)
Myopathy	2/21 (10 %)	1/11 (9 %)	8/94 (9 %)
Endocrine			
Impaired glycaemia/DM	1/21 (5 %)	3/11 (27 %)	8/94 (9 %)
Ocular			
Ptosis	0/21 (0 %)	0/11 (0 %)	3/94 (3 %)
Ophthalmoplegia	0/21 (0 %)	1/11 (9 %)	6/94 (6 %)
Optic atrophy	0/21 (0 %)	1/11 (9 %)	2/94 (2 %)
Retinitis pigmentosa	0/21 (0 %)	2/11 (18 %)	2/94 (2 %)
Other			
Short stature	1/21 (5 %)	1/11 (9 %)	1/94 (1 %)
Nephropathy	0/21 (0 %)	1/11 (9 %)	1/94 (1 %)

### Brain imaging

Magnetic resonance imaging was abnormal in 104 out of 117 patients scanned; a normal scan was reported in 13 patients; cerebellar atrophy was demonstrated in 92 patients. The remaining 12 abnormal scans showed predominantly small vessel ischaemia in keeping with age but no features to suggest mitochondrial disease. There were no differences on imaging between patients with and those without mitochondrial disease.

### Muscle analysis

Of the 126 patients with ataxia who underwent a muscle biopsy, 30 were identified on initial histopathological assessment as having features suggestive of mitochondrial disease. Three of these patients were subsequently found, on genetic testing, to have SPG7 and were therefore excluded from the mitochondrial group, leaving 27 patients in total with histologically suspected mitochondrial disease. Two additional patients were found to have mitochondrial disease on genetic testing despite normal muscle histology. Of these 29 patients, eleven had mitochondrial disease confirmed on further genetic analysis, either with the presence of mtDNA rearrangements or deletions (*n* = 3) or with the identification of specific mutations (m.8719G > A, p. Gly65* (MTATP6), m.9185 T > C, p. Leu220Pro (MTATP6), m.3243A > G (MTTL1), m.7510 T > C (MTTS1), c.811C > T, p.R271C/c.911G > A, pA304T (CABC1/ADCK3) as well as compound heterozygosity for two sequence variants in the POLG gene (c.1232 T > C, p. (Leu411Pro) and c.1721G > A, p. (Arg574Gln)). Thus the total number of patients with histologically suspected and/or genetically confirmed mitochondrial disease was 29/126 (23 %).

## Discussion

This retrospective review of a large number of patients (126) with progressive cerebellar ataxia who underwent muscle biopsy suggests that mitochondrial disease is likely to be a common cause of progressive cerebellar ataxia (up to 23 %) and that muscle biopsy is an important diagnostic test in such cases.

Patients with the ‘ataxia plus’ phenotype constituted the majority of patients referred for a muscle biopsy. 91 % of patients with histologically suspected/genetically confirmed mitochondrial disease had ‘ataxia plus’ as opposed to 9 % of patients with pure ataxia. Additional features such as deafness, impaired glycaemic control/diabetes, myoclonus, neuropathy or spastic paraparesis were therefore more likely to be associated with the diagnosis of mitochondrial disease.

Attention to the family history can be useful in interpreting muscle biopsy findings as well as directing and validating later genetic results. The presence of a family history and the mode of inheritance did not, however, predict the likelihood of mitochondrial disease in these patients. There are several possible reasons for this, including the late onset of the disorder in these families (and possible death from other causes in preceding relatives), and the recent onset of the mutations.

Genetic confirmation in patients with histological and histochemical changes suggestive of mitochondrial pathology was achieved in 9/27 (33 %) patients. In 2 patients a genetic diagnosis of mitochondrial disease was made even in the presence of normal histological changes in the muscle, suggesting that normal muscle histology and histochemistry does not exclude mitochondrial disease. It is also worth noting that patients with SPG7 can have muscle histological abnormalities suggestive of mitochondrial dysfunction, something that has been previously described [9]. For the remaining undiagnosed patients, the increasing availability of high-output whole exome sequencing will likely have a role in detecting pathogenic variants underlying ataxia syndromes [10, 11]. The use of muscle biopsy as a means of targeting downstream genetic analysis will expedite the identification of known mutations as well as providing evidence of a biochemical defect consistent with the genetic defect, thus adding weight to the presumed pathogenic nature of novel genetic variants.

At the time of review, 50 patients with normal muscle biopsies still had no specific diagnosis for their ataxia. Twenty-six of these patients (52 %) had a family history and presumed genetic ataxia is likely to be the cause. With the introduction of next generation sequencing a diagnosis was achieved in some of these patients as follows: 2 additional patients with SPG7, 2 patients with Niemann-Pick Type C [12], one patient with episodic ataxia type 2 [13], one patient with ataxia telangiectasia [14], one patient with ARSACS [15], 2 patients with SCA14 [16], one patient with SCA28 [17] and one patient with SCA35 [18]. If we are to exclude all the above patients where a diagnosis was eventually achieved the prevalence of mitochondrial disease becomes as high as 43 %. These findings emphasise the need for regular review and diagnostic re-evaluation of all patients with progressive ataxia, something that is the norm in UK ataxia centres.

## Methods

### Patient selection

Adult patients (>18 years) with unexplained cerebellar ataxia referred to a specialist ataxia clinic at either neuroscience centre (Sheffield or Newcastle) who underwent a muscle biopsy between 2004–2014 were included. Only those who had muscle biopsies to investigate progressive ataxia of undetermined cause following extensive investigations were included in this 10-year study period. Those with primary myopathy or other neurological features in the absence of cerebellar ataxia were excluded.

### Clinical evaluation

Clinical assessment and investigations were the same at both sites. A full history and examination was conducted allowing patients to be categorised as a ‘pure ataxia’ phenotype or an ‘ataxia plus’ phenotype (ataxia with associated features, e.g. peripheral neuropathy, epilepsy, diabetes). The additional clinical features were grouped according to systems (neurological, endocrine, ocular and other). Extensive investigations to determine the aetiology of ataxia were performed prior to the muscle biopsy. Such tests, depending on clinical indications, included blood cell counts, biochemistry, thyroid function, serology for coeliac disease, vitamin A, B12, E; metabolic screen for acyl-carnitines, urinary organic acids, very long chain fatty acids, phytanic acids, lysosymal enzymes, lipoproteins, serum electrophoresis, copper, caeruloplasmin, ferritin and iron amongst others. The presence of a family history including mode of inheritance was documented. Volumetric 3 T MR imaging of the cerebellum was undertaken in patients with cerebellar ataxia using a 3-T system (Philips ACHIEVA 3^.^0 T Best, Netherlands). The final diagnosis at the time of review was also noted.

### Genetic testing

Prior to performing a muscle biopsy, all patients had blood DNA genetic testing for common mitochondrial syndromes (MELAS, MERRF, NARP) and in selected cases when clinically indicated, mutations in the mitochondrial DNA polymerase gamma (*POLG*) gene. In addition, testing to exclude other ataxia syndromes was performed, including spinocerebellar ataxias (SCA1,2,3,6,7,12 and 17) and Friedreich’s ataxia (FA). Additional genetic tests including, for example, *DRPLA*, fragile X, ataxia occulomotor apraxia, episodic ataxias, *SPG7* screening were carried out if clinically indicated.

### Muscle biopsy

- Routine histopathology
  Muscle biopsies were performed using an open muscle (deltoid or quadriceps) biopsy technique. A portion of tissue was immediately frozen (liquid nitrogen cooled isopentane) in transverse orientation and cryosectioned (10 μm) in accordance with standard laboratory enzyme histochemistry and histology protocols. A second portion of tissue was stored at − 80 °C for molecular genetic studies. Routine histological stains used included haematoxylin and eosin (H & E), modified gomori trichrome and a stain for lipid (oil red O or sudan black); periodic acid Schiff (with and without diastase), nicotinamide adenosine dinucleotide–tetrazolium reductase (NADH–TR), cytochrome *c* oxidase (COx) and succinate dehydrogenase (SDH) histochemical reactions were undertaken. Histochemistry for COx and SDH were performed either as individual preparations or as a sequential reaction in which absence of COx reactivity in COx-deficient fibres allows the SDH tetrazolium reaction product to be visualised.
  Histological characterisation focused on identifying fibre size and type, architectural changes, signs of necrosis or regeneration, interstitial variations and immunohistochemical findings (in particular ragged red fibres, fibres with lipid accumulation and COx-deficient fibres). The percentage of COx-deficient fibres vs age was taken into consideration (COx-deficient fibres are extremely rare in normal individuals under the age of 50 years, therefore for patients >60 years of age we considered histochemical support of a mitochondrial aetiology if the frequency of COx-deficient fibres exceeded 2 % of the total biopsy) [19, 20]. Muscle biopsies obtained in Sheffield also underwent electron microscopy (EM) examination to look for additional evidence of mitochondrial disease such as abnormal mitochondrial accumulation, dysmorphic organelles and paracrystalline inclusions.
- Mitochondrial respiratory chain enzymes and mtDNA genetics
  Spectrophotometric measurements of individual respiratory chain enzyme activities and the matrix enzyme marker citrate synthase were performed as described [21]. Molecular genetic analysis was performed in all muscle biopsy samples showing histopathological signs of mitochondrial disease. Long-range polymerase chain reaction assays were used to screen for large-scale mitochondrial DNA (mtDNA) rearrangements [22], whilst in some cases sequencing of the entire mitochondrial genome was undertaken.

## Conclusion

- Mitochondrial disease is likely to be the cause of ataxia in up to 23 % of patients with otherwise idiopathic, progressive cerebellar ataxia. In such cases muscle biopsy is an important diagnostic test.
- Additional features such as deafness, impaired glycaemic control/diabetes, myoclonus, neuropathy or spastic paraparesis are more likely to be seen in the context of mitochondrial disease.
- Pure ataxia is rarely due to mitochondrial disease.
- Normal muscle histology and histochemistry does not exclude mitochondrial disease.
